# Precision control of polyurethane filament drafting and winding based on machine vision

**DOI:** 10.3389/fbioe.2022.978212

**Published:** 2022-09-16

**Authors:** Shilin Wu, Huayu Yang, Xiangyan Liu, Rui Jia

**Affiliations:** ^1^ Hubei Digital Textile Equipment Key Laboratory, Wuhan Textile University, Wuhan, Hubei, China; ^2^ School of Mechanical Engineering and Automation, Wuhan Textile University, Wuhan, Hubei, China

**Keywords:** PU filament, tube preparation, visual measurement, drafting control, winding control

## Abstract

In the biomedical field, polyurethane (PU) is widely used in interventional catheters, artificial hearts, artificial blood vessels, orthopedic materials, medical adhesives, and other medical devices. In this paper, a method based on machine vision was proposed to control the drafting and winding accuracy of PU filament in order to solve the problem of centrifugal runout when the mold rotates. The centrifugal runout of the mold directly affected the preparation efficiency and quality of long artificial blood vessel by wet spinning. Through non-contact real-time detection of the filament diameter and the angle between the axis of filament and the axis of mold, the motion parameters of the two motors driving the moving platform and the drafting roller could be adjusted in real time to achieve the purpose of online real-time control of filament drafting and winding accuracy. The vision control method proposed in this paper was used to carry out the PU tube preparation experiment. The visual measurement results of the filament diameter and the included angle were compared with the manual measurement results. The average value of the diameter error is 0.0096mm, and the average value of winding angle is 0.4777°. The results proved the accuracy of the visual measuring method and testified it feasible to using machine vision instead of manual method to detect filament diameter and winding angle. Properties of the prepared PU tube were tested and analyzed. The filament diameter measured by the 3D microscope was about 0.87 mm and significantly smaller than the filament diameter before winding. This indicated that the winding was uniform, the extrusion was tight, and the adhesion was good.

## Introduction

With the continuous research and development of polymer materials, their good performance has attracted extensive attention in the fields of mechanical engineering, textile engineering, aerospace, electronics, biomedicine, chemistry, military industry, architecture and so on. (Cheng and Zhao, 2017; Qu, 2018; Bao, 2019). In the biomedical field, the main applications of polymer materials are artificial organs, artificial tissues, and medical polymer materials. At present, biomedical polymer materials are divided into natural biopolymer materials and synthetic biopolymer materials. For example, cellulose and collagen are natural polymer materials, while PU, silicone rubber and polyethylene are high molecular materials prepared by chemical synthesis (Liang and Huang, 2016). The PU materials can obtain appropriate mechanical properties, wear resistance, elasticity, hydrophobicity, and other properties by adjusting the proportion of components. This makes the research and application of PU materials in the fields of cardiovascular system, urinary system, *in vitro* body surface and tissue repair have great potential value (Shao et al., 2022). The mechanical properties of PU could also be improved by chemical modification, so that it can be applied and studied in more fields (Wu et al., 2015). For example, Li et al. (2013) modified PU with boron nitride nanotubes to make boron nitride nanotubes (BNNT) PU composites with different volume fractions. Zhang and Su (2012) filled carbon nanotubes into PU to obtain composites with good mechanical properties. Wang et al. (2009) modified PU coating with nano ZnO to improve its anti-aging performance.

Since the 1980s, PU has been widely used in interventional catheters, artificial hearts, artificial blood vessels, orthopedic materials, medical adhesives, and other medical devices (Uscategui et al., 2018). The precision control of PU filament drafting and winding proposed in this paper is to prepare high-quality PU artificial blood vessels. At present, the clinical performance of large caliber (>6 mm) artificial blood vessels is good, while the clinical performance of small caliber (≤6 mm) artificial blood vessels is poor. The main problem is that the suture site of small caliber artificial blood vessel is easy to produce intimal hyperplasia which makes the lumen narrower and leads to thrombosis. Thrombus will affect the patency rate of blood vessels in the body, resulting in the shortening of the life of artificial blood vessels and the need for reoperation. Because of the low production efficiency and high cost of artificial blood vessels, many patients with vascular diseases missed the best treatment time (Wu et al., 2020). Therefore, in recent years, in order to prepare small caliber artificial blood vessels suitable for human body, scientific research workers have done researches on a variety of different materials and produced a series of artificial blood vessels of different materials, such as polyester artificial blood vessels, expanded polytetrafluoroethylene artificial blood vessels, silk artificial blood vessels and PU artificial blood vessels (Shaker et al., 2019).

Among them, PU artificial blood vessel is considered as the best material for making small caliber artificial blood vessel (Xu et al., 2012; Cheng and Fu, 2017), because of its excellent mechanical properties, biocompatibility, water permeability, fatigue resistance, and compliance matching with natural blood vessel. At the same time, its structural design and modification can further improve its performance in human body. In recent years, there are more and more methods to prepare artificial blood vessels with PU materials, such as electro-spinning (Christian et al., 2010), freeze-drying multi-step (Wang Q et al., 2004; Gao et al., 2014), spray phase transformation (Yaguchi, 2002), impregnation (Miyamoto et al., 2002; Pan et al., 2008), wet spinning (Zhang, 2015), etc.

Among them, the impregnation method, freeze-drying multi-step method and spray phase transformation method are more complex and time-consuming, which are not conducive to production. Electro-spinning method is similar to wet spinning method, which first prepares PU materials into filament and then winds them. However, compared with wet spinning method, electro-spinning method is more cumbersome, and it is not easy to improve the porosity of PU filament. Large porosity can accelerate the process of endothelial cell formation and improve the compliance of artificial blood vessels.

**FIGURE 1:**
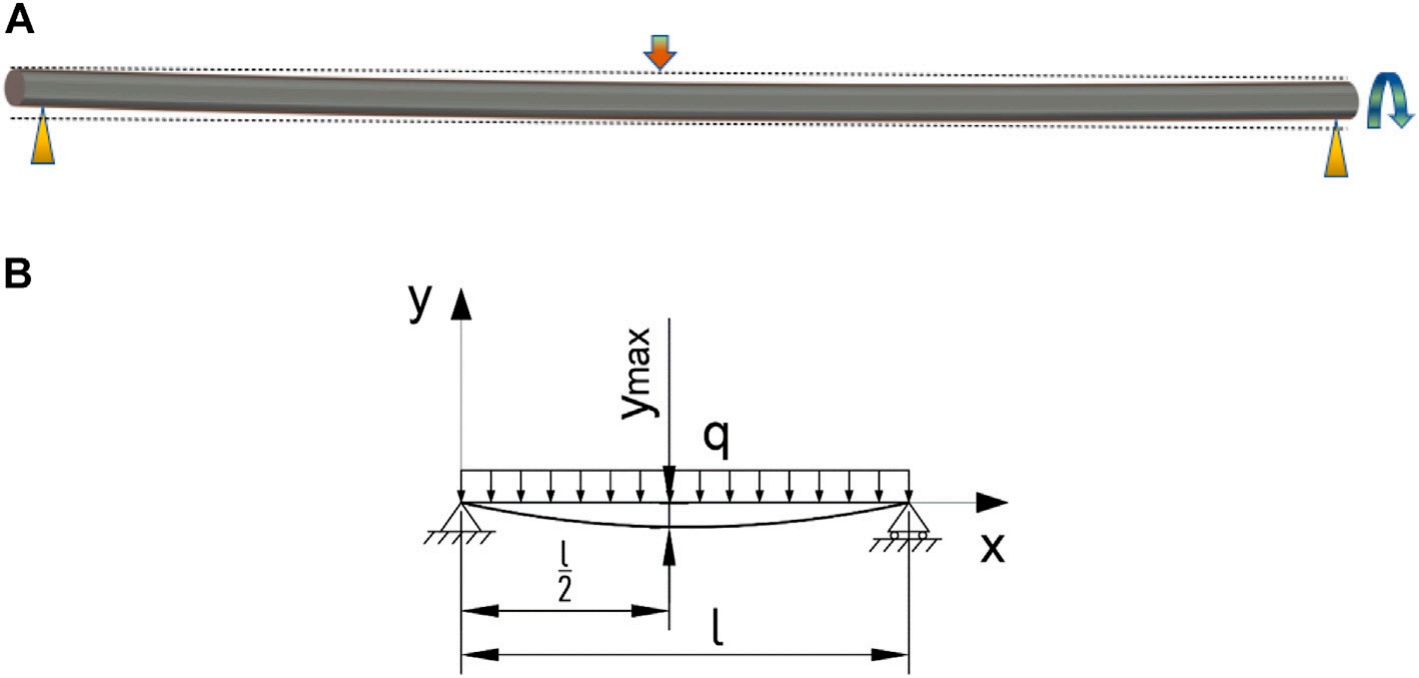
Diagram of mold deformation and force analysis. **(A)** Diagram of mold deformation. **(B)** Diagram of force analysis.

The tube preparation method studied in this paper was the wet spinning method. The conventional artificial blood vessel preparation is only a few hundred millimeters long. However, the total length of the PU tube prepared in this paper can reach 1400 mm. The mold used is a stainless-steel pipe with and outer diameter of 6mm, a wall thickness of 1 mm and a length of 2000 mm. Therefore, the overall weight of the mold is large and the length diameter ratio is large. Under the clamping conditions of support at both ends and suspension in the middle, the mold is an arc, as shown in Figure 1A.

When only the self-weight is considered, there is only the uniformly distributed load q generated by the self-weight on the mold, and the maximum deflection $y_{max}$ is at the center of the mold, as shown in Figure 1B. At a position of x on the mold, the deflection y can be calculated through the deflection equation $y=-\frac{qx}{24EI}\left(l^{3}-2lx^{2}+x^{3}\right)$ . Where, E is elastic modulus, I is the moment of inertia, l is the length of the mold, and x is the length of a certain section on the mold. The maximum deflection $y_{max}$ can be obtained by substituting the value of each quantity and $x=\frac{l}{2}$ into the above deflection equation, and $y_{max}=2mm$ . This explains the violent jumping of the mold in the process of high-speed rotation. And the faster the mold rotates, the more intense the jumping.

This directly affects the preparation efficiency and quality of PU tubes. Therefore, the machine vision detection technology was added to the PU filament preparation and PU corrugated tube integrated forming equipment involved in this paper, and used to monitor the forming process of PU tube in real time. The quality of the prepared PU tube was ensured by adjusting the motion parameters. The production efficiency of PU tube could be further improved in the case of unavoidable mold runout. The quality of the PU tube is also reflected in its overall uniformity and film-forming property. Film-forming property refers to the strength of the bonding property of the adjacent two coils of PU filament during the winding process. And the better the film-forming property, the better the mechanical property of the PU tube. There are certain requirements for the diameter and winding process of the PU filament, that is, the diameter of the PU filament should maintain a certain uniformity and the winding process shall ensure the close connection of adjacent filament coils.

**FIGURE 2:**
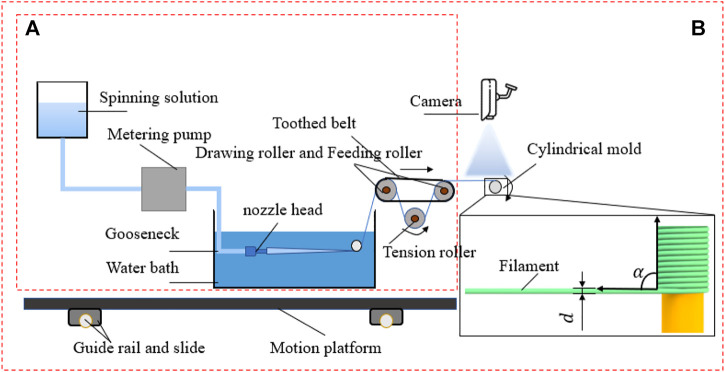
Schematic diagram of PU tube preparation process.

**FIGURE 3:**
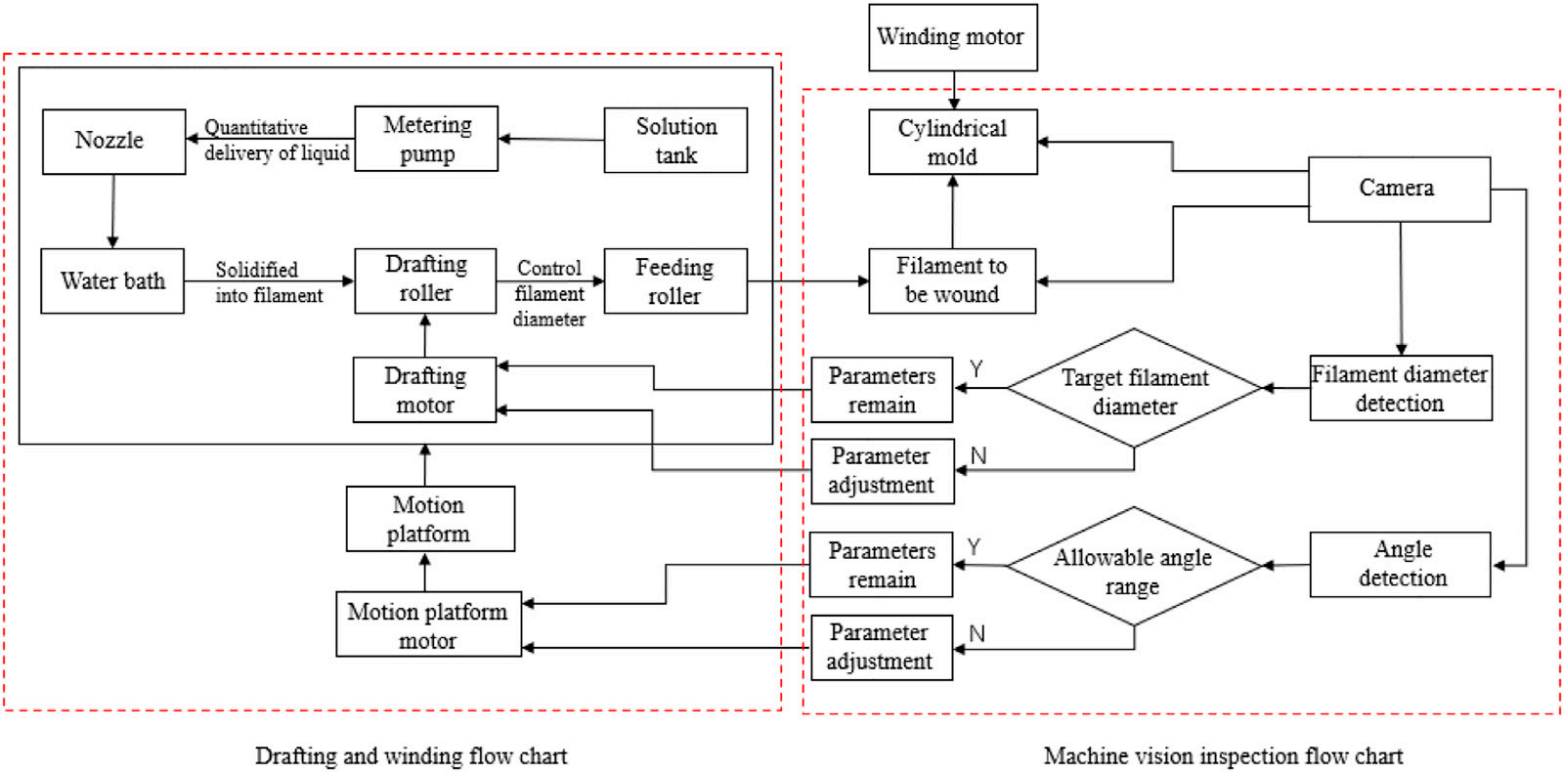
Block diagram of PU tube preparation and visual inspection.

In this paper, the preparation method of PU tube based on machine vision was proposed. The preparation flow and visual control diagram are shown in Figure 2 and Figure 3. Part A in Figure 2 mainly refers to the drawing part of PU.

Filament and Part B in Figure 2 refers to the visual inspection and winding part. The hardware facilities mainly include the storage tank of spinning stock solution, metering pump for quantitative solution delivery, small diameter spinneret, water bath, drafting mechanism, winding mechanism, visual camera, and motion platform. Figure 3 showed the preparation process, visual inspection, and target quantity to be controlled of the whole tube. The motion of the drafting mechanism was an important parameter that affected the diameter of PU filament (Wang Z. X et al., 2004; Zhao, 2019), while the motion parameters of the motion platform mainly affect the winding quality. The filament diameter and winding angle could be controlled only by controlling the speed of the drafting roller and the moving speed of the moving platform, to ensure the overall quality of the tube prepared. The winding angle is the included angle between the PU filament axis and the mold axis.

In the previous research, the control of filament diameter and winding angle were mainly observed by human eyes and adjusted according to experience, which led to unstable quality, low efficiency, and large error. It was impossible to directly measure the filament diameter and winding angle by contact method in the preparation process. Therefore, machine vision technology was adopted in the preparation process of PU tube. And the motion parameters were compensated and calculated through the algorithm to form a closed control loop and realize non-contact real-time control, as shown in Figure 3.

The non-contact measurement technology is mainly used in the measurement of high-risk environment, harsh environment, continuous moving objects, and vulnerable products. The existing non-contact measurement methods include electromagnetic induction method (Wu et al., 2013), laser detection method (Zhou, 2009; Li and Cao, 2020), prism free total station non-contact measurement method (Yao, 2020), machine vision detection method (Xiao et al., 2022), etc. The preparation process of PU tube is a high-speed movement process. The controlling of drafting and winding accuracy studied in this paper is completed synchronously in this process, so the machine vision method is more reasonable.

Machine vision which involves the interdisciplinary fields of computer science, image processing and pattern recognition, is an important branch of AI (Xie et al., 2022). It is known as the “eye of industry”, and it is a key technology used to replace the human eye to realize accurate detection, measurement, and control equipment (Liu and Yang, 2021; Zhu et al., 2022). Currently, the commonly used image processing libraries in machine vision include OpenCV, HALCON, MATLAB, VisionPro and LabVIEW.

Although VisionPro has a large market share in North America, its high runtime software license cost, lack of GPU processing and limited 3D visual algorithm library make it not favored by programmers. OpenCV is an open-source code library, which is inconvenient to debug, and its project development cycle is long. Although LabVIEW has high development efficiency, the accuracy and stability of the algorithm under its platform depend on image quality. HALCON is an image processing library under MVTec company in Germany. It has powerful 2D and full range 3D machine vision libraries. Its application scope covers positioning recognition, barcode/QR code recognition, measurement fitting, OCR tools, general visual inspection, defect detection, etc. The price of runtime license is greatly reduced. HALCON provides higher bit depth image processing and supports common operating systems and embedded devices, which makes more and more machine vision practitioners use HALCON to complete projects.

The machine vision related work completed in this paper mainly includes camera calibration, image acquisition, image processing and feature extraction. The calibration template provided by HALCON was used in the calibration. And the radial distortion of the lens and the solution method (Gao et al., 2020) are considered. The function library of HALCON was given full play to, which improved the calibration accuracy and calculation efficiency. And it effectively monitored and regulated the drafting and winding process in the process of PU tube preparation, ensuring the stability of tube preparation, providing the technical support for improving the preparation efficiency of artificial blood vessels, and the reference for the wet spinning process and detection of other materials.

## Visual measure of filament diameter and included angle

**FIGURE 4:**
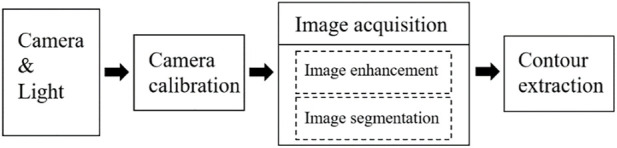
Flow chart of visual inspection.

In the process of PU spinning and winding, the drafting mechanism could control the filament diameter, and the moving speed of the moving platform could determine the angle between the filament and the mold. In the actual preparation process of PU tubes, it was found that if all parameters were set to constant values, the filament diameter would slowly increase with the passage of time, and the increase of filament diameter would also change the angle between the filament and the mold, resulting in poor quality of PU tubes or the preparation failure. In this paper, the machine vision was adopted to detect the filament diameter as well as included angle in real time and the differences between the target values were calculated to obtain new parameters. These new parameters were sent to the control motor, to achieve the purpose of controlling the filament diameter accuracy and winding accuracy. The overall visual inspection flow was shown as Figure 4.

### Camera and light source

Machine vision system is basically composed of the camera and light source. The camera is equivalent to the eye. The light source is the key to ensure that the camera can capture the image. A suitable light source directly affects the quality of acquired images, reduces the possible noise in image, reduces the difficulty of image recognition, and improves the positioning and detection accuracy of the machine vision system.

The camera used in this paper is an Intel RealSense depth camera d435i camera. Its main functional parameters are shown in Table 1.

**TABLE 1:** Camera function parameter table.

Parameter	Specification
Depth field of view	85.2 ° × 94 °×58°( ± 3°)
Depth stream output resolution/frame rate	highest1280 × 720/90fps
Depth range	0.1–10 m
RGB sensor resolution/frame rate	1920 × 1,080/30fps
RGB sensor FOV	69.4 ° × 88 °×42.5°( ± 3°)
Camera size	90 × 25×25 (mm)
Connector	USB 3.0 Type-C

Several common light sources were compared and white LED lamp was selected as the light source in this paper. LED lamp has many kinds of colors, long working life and various shapes, and it can meet different working environments. Fluorescent lamp is cheap, but it has a lot of heat. Xenon lamp has a short working life and a lot of heat. The heat of the light sources has a certain impact on the filament. The cost of halogen lamp is low, but the intensity is insufficient, which affects the effect of image acquisition. Specific properties of common light sources are listed in Table 2.

**TABLE 2:** Specific properties of Common light Sources.

Light source	Colour	Working life/h	Luminance	Characteristic
LED light	Red/yellow/green/white/blue	6000–10000 h	Lighter	Solid, multi profile
Fluorescent lamp	White, light green	5000–7000 h	Bright	Hot, cheap
Halogen lamp	White, light yellow	5000–7000 h	Highlight	Low cost and low luminous rate
Xenon lamp	Blue white	3000–7000 h	Bright	Hot

### Camera calibration

Camera calibration is the process of determining the internal and external parameters and distortion parameters of the camera. The distortion of camera lens is the general term of the inherent perspective distortion of optical lens. In the process of image acquisition, there is produce distortion when the path points pass through the camera lens, resulting in the geometric distortion of the image. The distortion cannot be eliminated, so it is necessary to calibrate the camera to correct the distortion. Whether it is image measurement or machine vision application, the calibration of camera parameters is very important. The accuracy of calibration results and the stability of algorithm directly affect the accuracy of machine vision systems. There are many camera calibration methods, and the camera calibration function of HALCON is suitable for many occasions. And it was the calibration method adopted in this paper.

**FIGURE 5:**
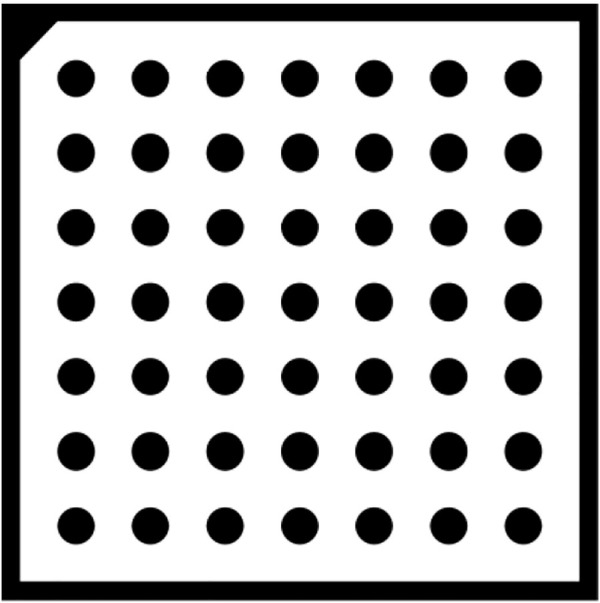
7 × 7 dot calibration plate.

When calibrating the camera, the operator gen_caltab in HALCON is used to generates a calibration plate with seven rows and seven columns, and the distance between the centers of circles is 12.5 mm. The distance between the centers of two adjacent circles is twice the diameter of a single dot, so that the size of the calibration plate is 1/3 to 1/2 of the camera’s field of view, as shown in Figure 5. The calibration steps contain: loading 15–20 images of the calibration board through the offline image loading method, selecting the image with the most appropriate position as the reference pose, calibrating to finally obtain the internal and external parameters of the camera. The specific calibration result data were shown in Table 3.

**TABLE 3:** Camera parameters& Camera pose.

Contents	Parameter names	Date
Camera Parameters	Cell Width (Sx)	7.68037 μm
	Cell Height (Sy)	8.3 μm
	Focal Length	1.22837 mm
	Kappa	620.769 1/ $m^{2}$
	Center Column (Cx)	249.752 pixels
	Center Row (Cy)	353.746 pixels
	Image Width	1,280 pixels
	Image Height	720 pixels
Camera Pose	X	147.055 mm
	Y	-4.32128 mm
	Z	35.4113 mm
	Rotation X	0.136,939°
	Rotation Y	356.56°
	Rotation Z	303.543°

### Image acquisition

**FIGURE 6:**
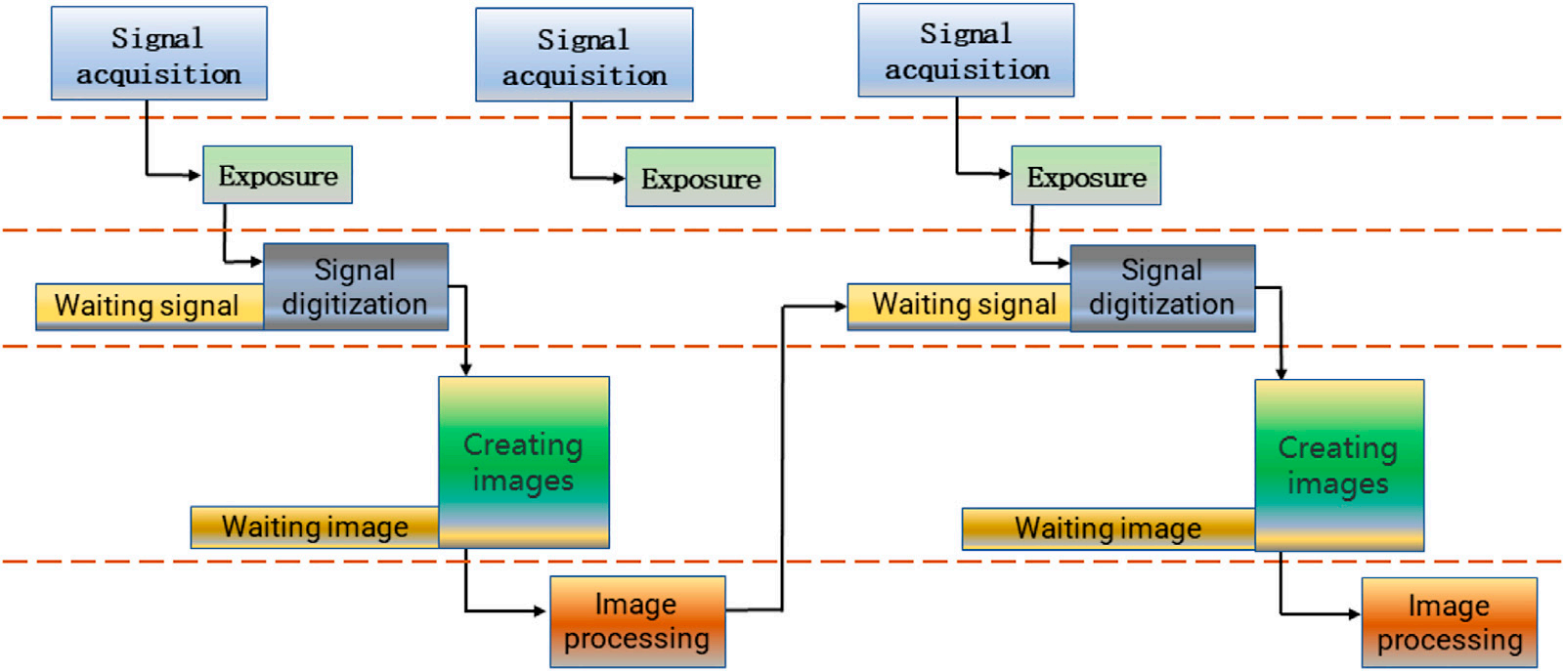
Flow chart of Synchronous acquisition.

Image acquisition is an important part of machine vision system. It converts the main part and characteristics of visual image into a series of data which can be processed by computer. In the HDevelop development environment of HALCON, there are two image acquisition modes: synchronous acquisition and asynchronous acquisition. In the process of synchronous acquisition, image capturing and image processing are carried out in sequence. After image capturing, Handle of image is generated and then image processing is carried out. After image processing, the next frame of image is captured after waiting for the next acquisition instruction. The specific process is shown in Figure 6.

However, in the process of synchronous acquisition, the working mode is to send the acquisition signal according to the frequency of the frame rate set by the camera. This may cause the signal of the next frame to be missed after the image processing is completed, and the image of the middle frame may be also lost. In this case, the actual frame rate may not reach the theoretical frame rate set by the camera.

**FIGURE 7:**
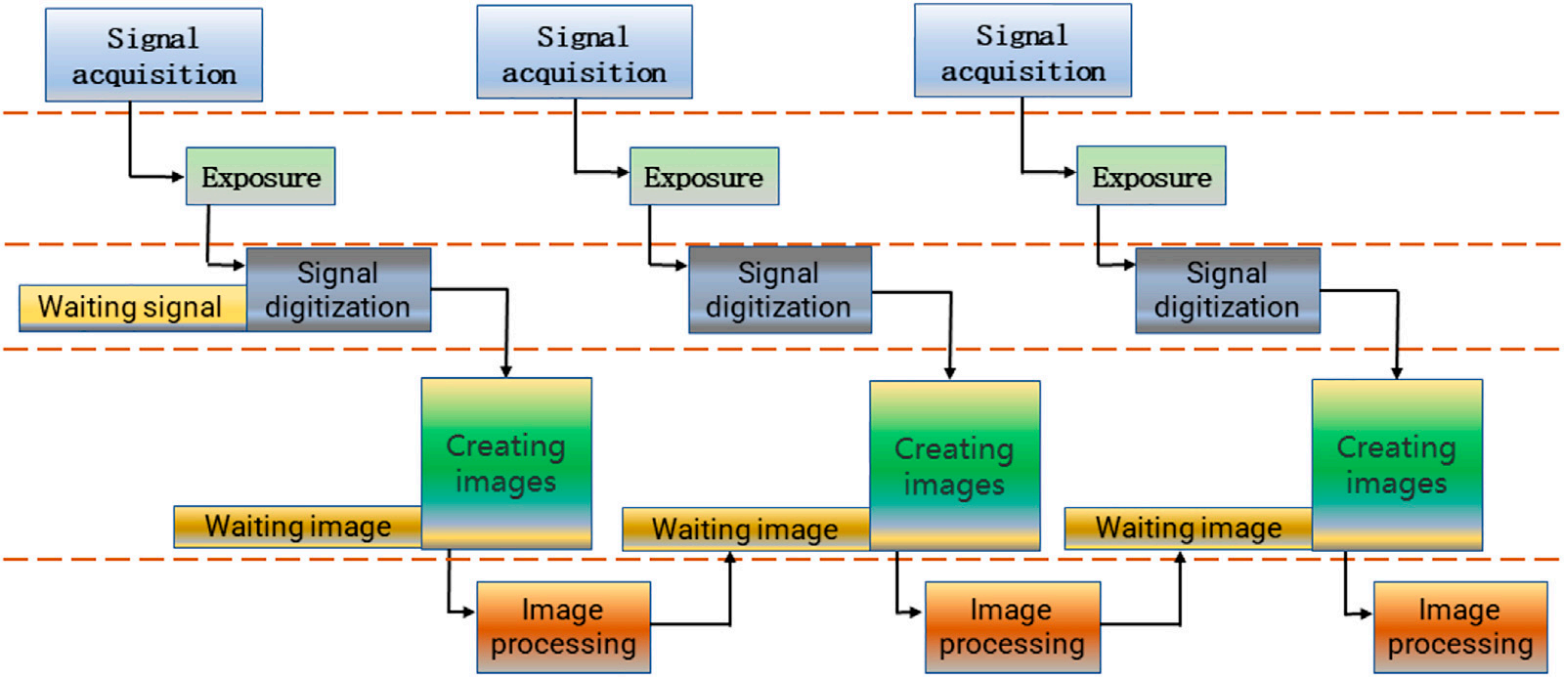
Flow chart of asynchronous acquisition.

In the asynchronous acquisition operation, the two steps of image capturing and image processing are separated, and can be operated asynchronously. To put it simply, the next frame of image can be captured while processing the present image. The flow diagram is shown in Figure 7. Asynchronous acquisition is the same as the first step of synchronous acquisition. The difference is that after the received image is collected asynchronously, the acquisition handle directly acquires the next frame image, and the work of the image processing operator is to process the previous frame image and continue to process the next frame image. After the processing is completed, it continues to call asynchronous acquisition until all images are acquired. In this way, the full frame rate can be achieved without frame leakage. The dual cache strategy is required when using asynchronous acquisition, because there are two different storage areas for storing and processing images and capturing images.

The asynchronous acquisition method was selected to acquire the required images, considering the characteristics of the above two image acquisition methods and combining with the needs of the real-time detection feedback of drafting and winding proposed in this paper.

### Image processing

In this paper, it is necessary to detect the image edge according to the requirements of filament diameter detection. In the aspect of detail enhancement, the second-order differential is better than the first-order differential.

And it is an ideal feature suitable for image sharpening. Using second-order differential Laplacian to sharpen the image is a common method. A spatial sharpening filter can be obtained through constructing a filter template based on the second-order differential discrete formula. This algorithm subtracts the second derivative of the gray value of the current position from the gray value of all positions of the image. Through this algorithm, the small gray value in the gray value of the edge position will become smaller, the large gray value will be larger, and the second derivative of the gray value of other positions will be zero, so this operation will not affect the gray value of the image.

A two-dimensional image expressed by Laplace operator can be expressed as:

$$\nabla^{2}f=\frac{\partial^{2}f}{\partial x^{2}}+\frac{\partial^{2}f}{\partial y^{2}}$$ (1)

Since the *x* and *y* directions need to be considered for image sharpening, when the position in the *x* direction is fixed, the second derivative of the gray level of the image in the *y* direction is:

$$\frac{\partial^{2}f}{\partial y^{2}}=f\left(x,y+1\right)+f\left(x,y-1\right)+2f\left(x,y\right)$$ (2)

When the position in the *y* direction is fixed, the second derivative of the gray level of the image in the *x* direction is:

$$\frac{\partial^{2}f}{\partial x^{2}}=f\left(x+1,y\right)+f\left(x-1,y\right)-2f\left(x,y\right)$$ (3)

When both *x* and *y* directions are considered, the second derivative of the image gray level, that is, the 2D Laplace operator of the image, is expressed as:

$$\nabla^{2}f\left(x,y\right)=\frac{\partial^{2}f}{\partial y^{2}}+\frac{\partial^{2}f}{\partial x^{2}}$$

=f(x+1,y)+f(x−1,y)+f(x,y+1) (4)

+f(x,y−1)+f(x,y−1)−4f(x,y)               

The Laplace operator to enhance the image can be written as:

$$g\left(x,y\right)=f\left(x,y\right)+c\left[\nabla^{2}f\left(x,y\right)\right]$$ (5)

**FIGURE 8:**
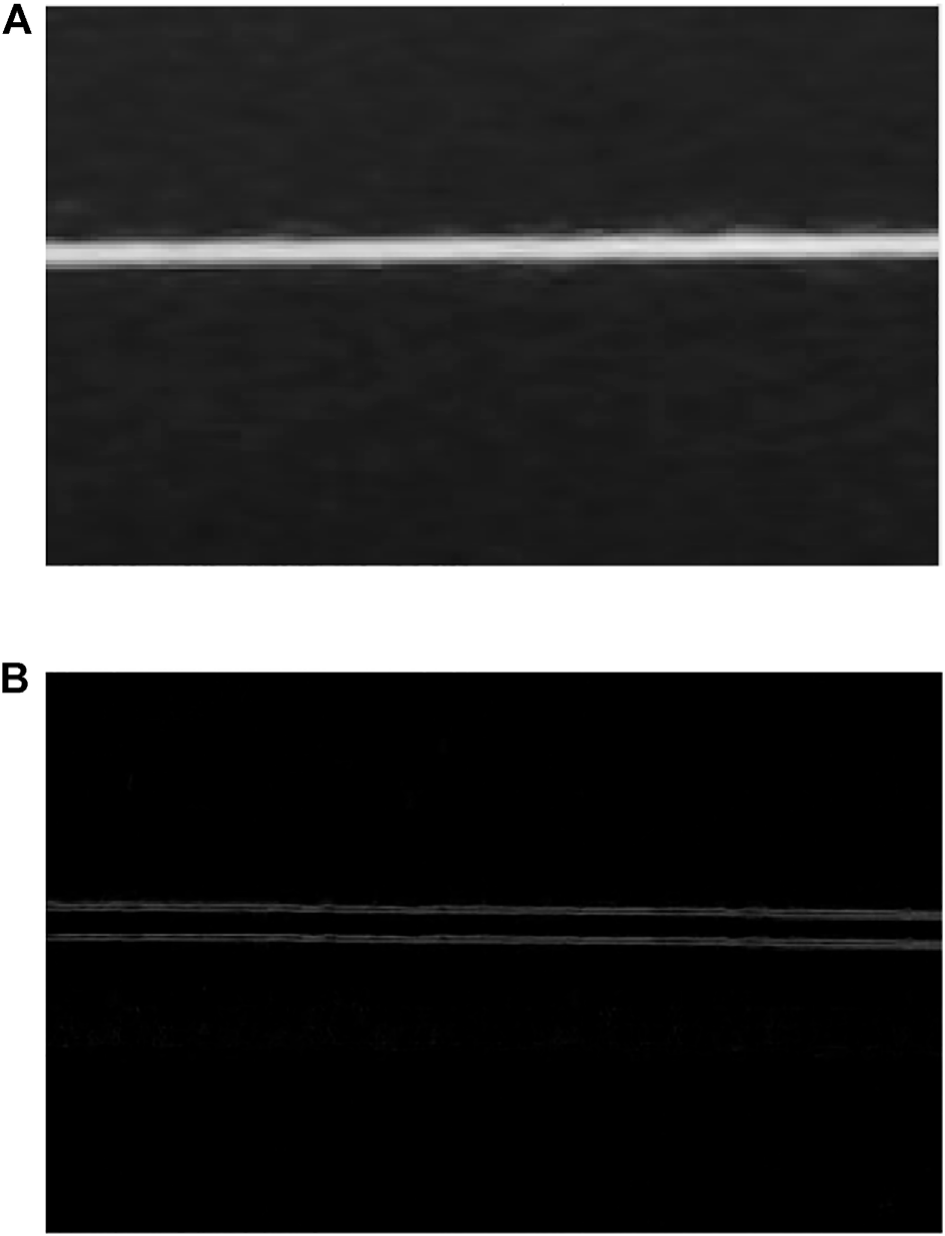
Discrete Laplace Filtering. **(A)** Original image. **(B)** Filtered image.

Where c represents a constant, and the value is one or -1. g(x,y) and f(x,y) represent the sharpened image and the original image respectively. The actual application effect of the discrete Laplace filter is shown in Figure 8. Figure Figure8A shows the original image of the filament. There is obvious noise at the edge and the whole image is fuzzy, which is not conducive to the subsequent calculation of the diameter of the filament. After filtering, the outer contour of the filament is clear, which is conducive to the calculation of the diameter of the filament, as shown in Figure 8B.

Image segmentation divide the image into several disjoint regions according to the characteristics of gray, color, spatial texture, and geometric shape, so that these features show consistency or similarity in the same region and obvious differences between different regions, to separate the target from the background. There are four general image segmentation methods, which are based on threshold, edge, region, and graph theory respectively. In this paper, a threshold-based segmentation method was used to segment the image. The image segmentation method based on threshold divides the gray threshold according to the gray characteristics, then compare the gray value with the gray threshold, and divide each pixel into regions. The threshold-based binarization process of an input image can be expressed as:

$$g\left(x,y\right)=\{\begin{array}{c} 1,f\left(x,y\right)\geq T\\ 0,f\left(x,y\right)<T \end{array}$$ (6)

Where T represents the threshold. When the gray value is greater than or equal to T, the value of this pixel is 255, which is the image point we need. When the gray value is less than T, the value of this pixel is 0, which is treated as background point.

In the actual winding detection, the gray difference between the winding filament, the rotating mold and the background is very large. Using the threshold operator in HALCON to segment the image is simple and fast. This operator can be expressed as:

$$\text{R}\prime =\left\{\left(\text{x},\text{y}\right)\in \text{R}|g_{\text{min}}\leq \text{g}\left(\text{x},\text{y}\right)\leq {\text{g}}_{\text{max}}\right\}$$ (7)

Where  g(x,y) represents the part of the original image that needs to be retained, R′ represents the image area after threshold segmentation, and  R  represents the original image area. Pixels whose gray value is greater than $g_{min}$ and less than $g_{max}$ are selected into R′ .

**FIGURE 9:**
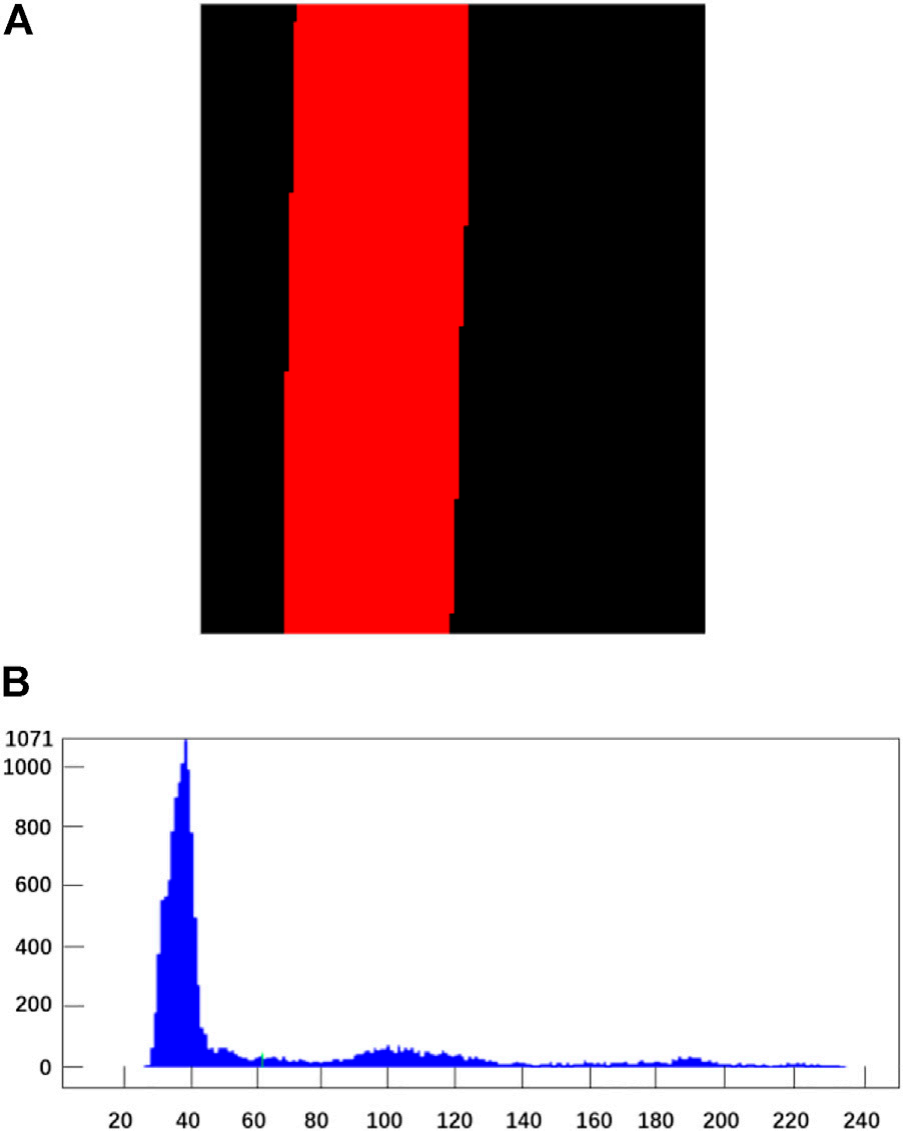
image Segmentation processing diagram of winding mould. **(A)** Original image. **(B)** Gray histogram.

Figure 9 showed the effect picture and gray histogram of the winding mold after image segmentation using the threshold operator.

### Contour Extraction and angle calculation

**FIGURE 10:**
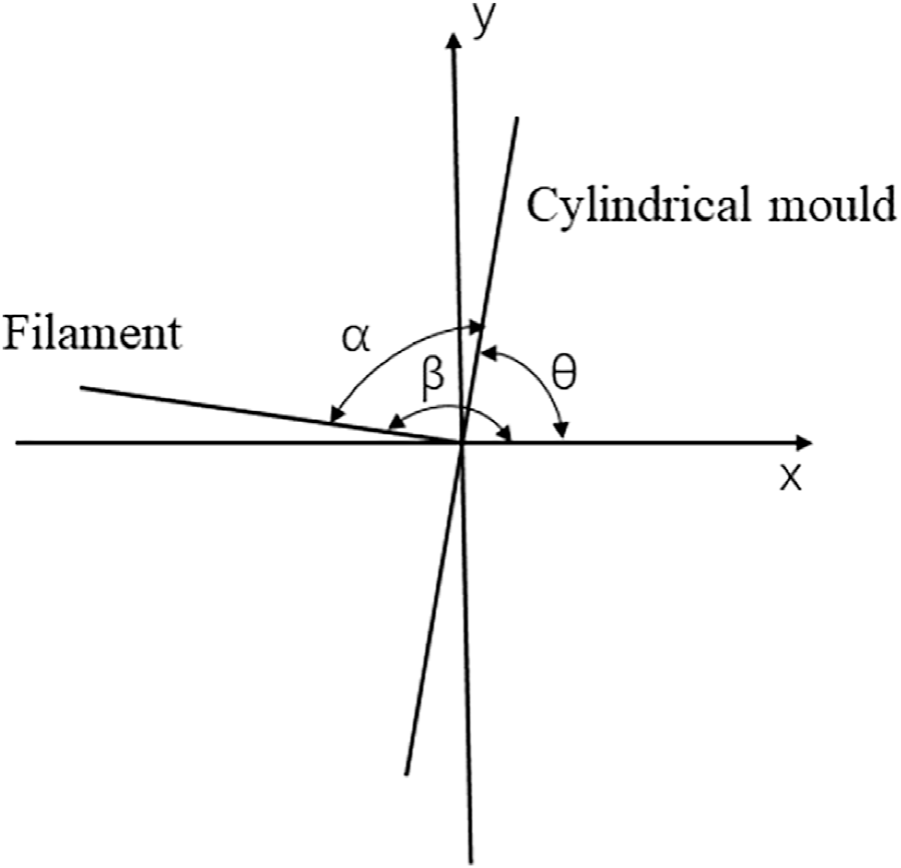
Diagram of winding angle.

For controlling the drafting and winding of PU filament, it is necessary to detect the included angle α and filament diameter d first, and then calculate the difference with the target value, to carry out feedback control. However, the included angle calculated directly by the equation is not accurate. Especially, when the mold is constantly jumping, the image processing is greatly disturbed, which affects the accuracy of the result. Therefore, an indirect method was used to solve the included angle α. As shown in Figure 10, the X-Y plane coordinate system is established. The *Y* axis direction is the mold axis, and the *X* axis direction is the filament axis which is also the drafting direction. As the mold itself is a 2000 mm long and 6 mm diameter stainless-steel pipe, its self-weight is large and its rigidity is insufficient. Thus, during high-speed rotation, it is easy to generate large centrifugal force which makes the mold jump obviously. Therefore, its actual motion posture is at a certain deflection angle θ(θ ≠ 90°) with the *X* axis, and the farther the deflection angle is from the mold support point, the greater the deflection angle. The included angle between the winding filament and axis X is β, the included angle with the mold is α, and the included angle α=β−θ .

**FIGURE 11:**
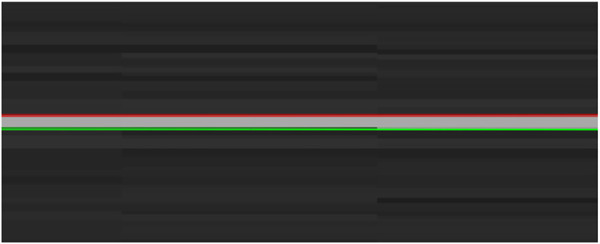
Contour extraction of Filament.

In the process of solving the diameter d of the filament, HALCON operator “edges_sub_pix” is used to extract sub-pixel edges from the images acquired by the machine vision system, to obtain the edge contours on both sides of the filament. Then, the operator “fit_line_contour_xld” is used to fit the two contours respectively to obtain two straight lines: a red line, and a green line, as shown in Figure 11. Finally, the average value is calculated as the final output by collecting multiple groups of points in the vertical direction of two straight lines and calculating the distance by the “distance_ss” operator, to obtain the diameter d .

## Control of drafting and winding

### Control of drafting

There are two difficulties in the preparation of PU tubes. Namely, the control of filament diameter and the control of filament winding tightness. According to the motion analysis of the drafting mechanism in the schematic diagram of tube preparation, the control of the filament diameter is closely related to the speed of the drafting roller and the extrusion amount of the metering pump. In order to simplify the control process and improve the calculation efficiency and accuracy, the method of controlling variables was adopted to make the speed difference between the solution extrusion amount and the output amount of the filament, to control the diameter of the filament. The equation of the filament diameter can be written as:

$$d=\frac{Q}{{\text{n}}_{1}{\text{πd}}_{1}}$$ (8)

Where  d is the diameter of the filament, in mm, *Q* is the extrusion flow of the metering pump, in mm ^3/s, $d_{1}$ is the diameter of the drafting roll, in mm, and $n_{1}$ is the speed of the drafting roll, in r/s.

In the actual drafting process, the diameter of the drafting roll $\text{er}\;d_{1}$ and target filament diameter  d was determined. In order to facilitate control, the extrusion flow *Q* was also taken as a fixed value, so that the theoretical filament diameter could be calculated only by controlling the speed $n_{1}$ of the drafting roller. However, the runout of the mold during high-speed rotation caused the calculated filament diameter to be inconsistent with the actual measured value, so it is necessary to detect the filament diameter in real time through machine vision and feedback the difference, to adjust the speed $n_{1}$ of the drafting roller motor to keep the filament diameter within the target value range.

### Control of winding

**FIGURE 12:**
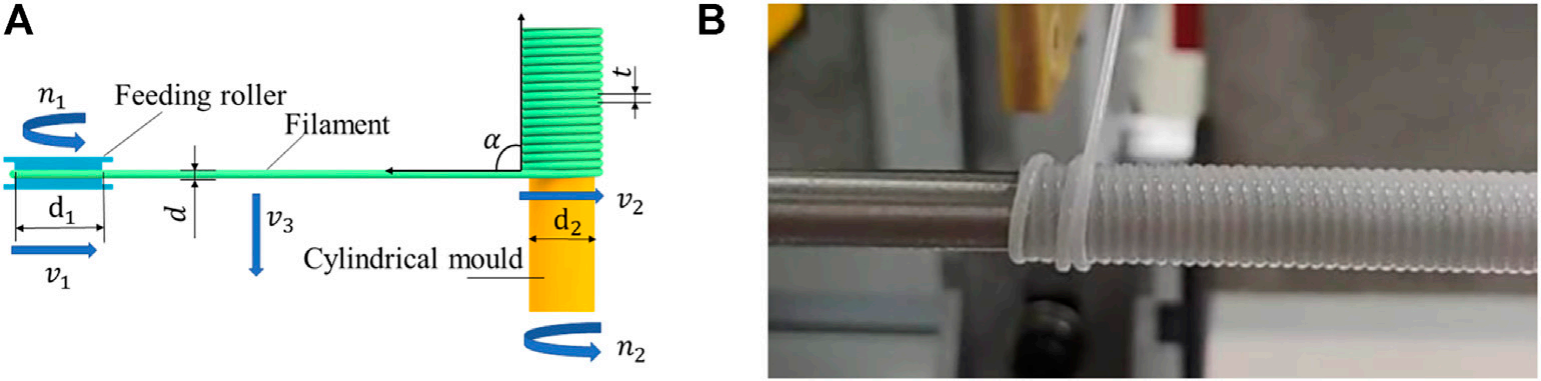
Winding diagram and winding diagram. **(A)** Winding Diagram (Top View). **(B)** Reverse Winding Diagram.

According to the analysis of winding motion in the schematic diagram of PU tuber preparation, the tight winding of filament on the mold was completed under the joint action of the mold rotation and the horizontal movement of filament on the moving platform. The schematic diagram of the process was shown in Figure 12A.In the figure, $n_{1}$ was the speed of filament feeding roller, $n_{2}$ was the mold speed, $v_{1}$ was the linear speedof filament feeding roller, $v_{2}$ was the linear speed of the mold, $v_{3\;}$ was the horizontal moving speed of the moving platform, d  was the diameter of filament, $d_{1}$ as the diameter of filament, $d_{2}$ was the mold diameter, α was the included angle between the filament and the mold.

t was the pitch of adjacent filament coils. Unit of speed is r/s, unit of linear speed is mm/s, unit of diameter is mm and unit of the angle is (°)。

According to the winding process, during the winding process, the filament was required to keep a small amount of stretching, so that the filament could closely fit the surface of the mold when it was wound on the mold. Therefore, the mold rotation speed $\;v_{2}$ should be greater than or equal to the linear speed $v_{1}$ of the filament feeding roller. The centrifugal force runout had a direct impact on the filament tension when the mold rotated. At the same time, in order to ensure the film formation of PU tube, the filament needed to be closely connected during the winding process. So, the included angle  α should be an acute angle to enable the filament to fall from the previous coil during the winding process, to achieve the purpose of close connection between the filaments.

As the included angle was too small, the filament would wind back, as shown in Figure 12B. So, according to the actual experimental experience, the angle was controlled within 78°–88°, and the tightness between the filament coils was good. Included angle α was controlled by adjusting the moving speed $v_{3}$ of the mobile platform. When $v_{3}$ was slightly less than the axial increment speed $v_{4}$ of filament winding, the included angle α would gradually become an acute angle. $v_{4}$ was the increasing speed of filament in the winding axial direction and its theoretical value equals $n_{2}d$ . When α was within the range of 78°–88°, $v_{3}$ was adjusted to be consistent with the axial increment speed $v_{4}$ .

At the beginning $,v_{3}<v_{4}$ , when α reached the appropriate range, adjusting $v_{3}$ to make $v_{3}=v_{4}$ , so that α remain unchanged. Factors influencing $v_{3}$ are same as that of $v_{4}$ , i.e., filament diameter d and mold speed $n_{2}$ . The relationship between them can be expressed by Eq. 9.

$$v_{3}=n_{2}d$$ (9)

In the same way, due to the runout of the mold and the extrusion deformation of the filament when they were closely connected, *t* is smaller than d . Therefore, the calculated value of Eq. 9 was a theoretical value, which required real-time adjustment according to machine vision detection result. The horizontal movement of the motion platform was transformed from the rotation of the motor through the ball screw. The relationship between $v_{3}$ and the lead of screw ball can be expressed by Eq. 10.

$$v_{3}=n_{3}p$$ (10)

Where, $n_{3}$ is the motor speed of the moving platform, and p is the lead of the ball screw.

Combining Eq. 9 and Eq. 10, the controlling variable $n_{3}$ could be directly obtained, and it could be written as:

$$n_{3}=\frac{n_{2}d}{p}$$ (11)

To sum up, when the mold speed $n_{2}$ , target filament diameter d and the ball screw lead p were the determined values, the motor speed of the moving platform could be calculated, and the filament winding process could be successfully completed through the adjustment of $v_{3}$ .

## Experimental results and analysis

### Visual detection results and analysis

In the process of tube preparation experiment, the winding filament diameter $d_{1}$ and winding angle α were real-time visual inspected. The visual measurement results of $d_{1}$ and α were compared with the manual measurement results. The data were shown in Table 4, in which 10 groups of data were compared. And the data were also statistical analyzed. Their means, variances, and standard deviations (SD) of the measured values of those two methods were also listed in Table 4. From the statistical results, it could be seen that the visual method was more stable and accurate than the manual method.

**TABLE 4:** Measured results of filament diameter and included angle.

Number	Angle (°)		Diameter (mm)	
	Vision	Manually	Vision	Manually
1	87.053	86.55	1.042	1.04
2	87.835	87.45	1.046	1.04
3	87.356	87.05	1.064	1.04
4	87.533	86.55	1.052	1.06
5	87.782	88.50	1.065	1.06
6	87.158	87.20	1.052	1.06
7	87.355	87.85	1.055	1.04
8	87.442	87.30	1.050	1.04
9	87.556	86.40	1.047	1.04
10	87.753	87.80	1.051	1.04
Mean	87.482	87.265	1.052	1.046
Variance	0.0687	0.4456	5.404e-05	9.333e-05
SD	0.2622	0.6675	0.0074	0.0097

It could be found by analysis that the average value of the diameter error was 0.0096mm, the average value of the winding angle error is 0.4777°. The results proved the accuracy of the visual measuring method and proved it feasible to using machine vision instead of manual method to detect filament diameter and winding angle.

### Performance test and analysis of finished tube

**FIGURE 13:**
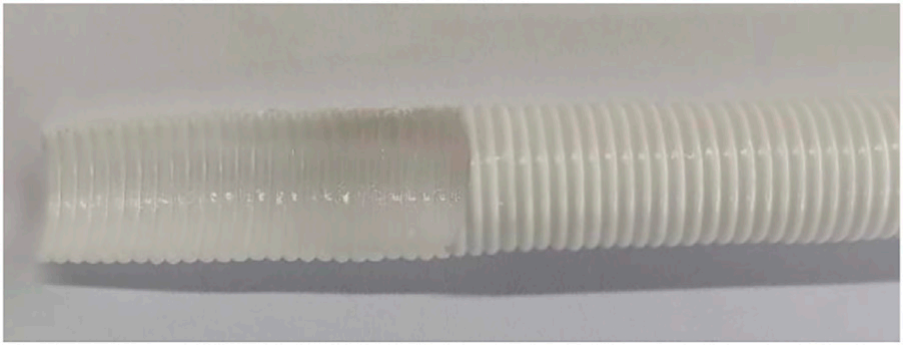
prepared PU tube.

The prepared PU tube was shown in Figure 13. The inner and outer appearance of the tube were relatively uniform, without cracks, with good shape retention, indicating the filament coils were closely bonded.

**FIGURE 14:**
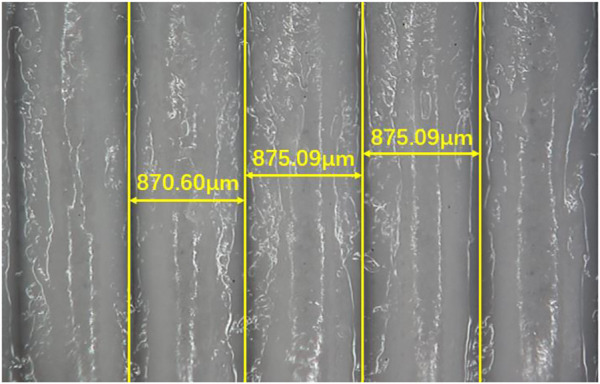
Inwall micrograph of prepared PU tube.

Micrograph of the prepared PU tube is shown in Figure 14. From the marked yellow dimension line, the measurement results were 870.60μm, 875.09μm and 875.09 μm respectively, which indicated the filament diameter uniformity of the winding filament was good, and the drafting process was stable. The diameters were significantly smaller compared with the filament diameters measured in Table 4, indicating the filaments were tightly extruded during the winding process, with good adhesion. And there was an obvious filler that was PU film in the gap between the filament coils, which indicated that the film-forming effect was good.

## Conclusion

In this paper, a method of drawing and winding precision control for PU filament based on machine vision was proposed. This method was used to carry out the PU tube preparation experiment. The visual measurement results of the filament diameter and winding angle during the preparation process were compared with the manual measurement results and the properties of the prepared PU tube were tested and analyzed. The comparison and analyzation results proved the feasibility and accuracy of the vision measurement method and the effectiveness of the vision control method.

In the next work, we will further verify the feasibility and stability of the visual control method, try to reduce mold runout and difficulty of visual processing in terms of hardware, and improve the calculation efficiency and control accuracy.

## Data Availability

The original contributions presented in the study are included in the article/Supplementary Materials, further inquiries can be directed to the corresponding author.
